# Flap-making patterns and corneal characteristics influence opaque bubble layer occurrence in femtosecond laser-assisted laser in situ keratomileusis

**DOI:** 10.1186/s12886-022-02524-6

**Published:** 2022-07-11

**Authors:** Xi He, Shi-Ming Li, Changbin Zhai, Li Zhang, Yue Wang, Xiumei Song, Yi Wang

**Affiliations:** Beijing Tongren Eye Center, Beijing Tongren Hospital, Capital Medical University; Beijing Ophthalmology & Visual Sciences Key Laboratory, Beijing, 100730 China

**Keywords:** Opaque bubble layer, Risk factors, FS-LASIK

## Abstract

**Background:**

Opaque bubble layer (OBL), which generates from photo-disruptive procedures on the cornea, has been a common phenomenon during femtosecond laser-assisted refractive surgeries and it would potentially impact eye tracking and flap lifting. And we have observed that an updated flap-making pattern could form less OBL clinically than the traditional pattern, which needed further approval. Thus, the purpose of this study is to prove our observation and investigate the possible risk factors related to the occurrence and type of OBL in laser in situ keratomileusis (LASIK) flaps using the Visumax laser system.

**Methods:**

This prospective study included 167 eyes of 86 patients (mean age: 27.5 ± 6.1 years) undergoing bilateral femtosecond laser-assisted laser in situ keratomileusis (FS-LASIK) for myopia/myopic astigmatism by the same surgeon from April 2020 to August 2020. Preoperative data on refraction, central corneal thickness (CCT), and keratometry as well as intraoperative data were included for analysis. A new flap-making pattern creating an offset between flap-cut and side-cut was adopted to compare with the traditional pattern. The operation video of flap formation was analyzed to identify the existence and type of OBL. The area covered by OBL and the ratio of OBL to flap were calculated using Image J software.

**Results:**

Among 167 eyes, 54 eyes (32.3%) developed OBLs, consisting of 31 as hard OBL coexisting with soft OBL, and 23 as soft OBL alone. The OBL incidence was significantly reduced in eyes with the new flap-making pattern compared with the traditional pattern (13.8% vs. 52.5%, *P* < 0.001). Hard OBLs had larger area ratios than soft OBLs (14.3 ± 8.3% vs. 1.1 ± 1.8%, *P* < 0.001). Univariate analyses revealed that eyes with more myopia, thicker CCT, and traditional flap-making patterns were more likely to develop OBLs. Multivariate analysis further confirmed that more myopia, thicker CCT, and traditional flap-making pattern were risk factors for OBLs. A Larger corneal diameter was associated with a higher incidence of hard OBL when applying the traditional flap-making process.

**Conclusion:**

More myopia, thicker CCT, and larger corneal diameter were risk factors for OBL development during flap creation, whereas a flap-making pattern with an offset between flap-cut and side-cut could reduce the incidence of OBL.

## Background

Refractive surgeries have now been widely applied in correcting myopia and astigmatism. Femtosecond assisted laser in suit keratomileusis (FS-LASIK) is one of the main surgical approaches nowadays, it has been nearly two decades since the first FS-LASIK was performed [1]. Flap creation using femtosecond laser allows designing flaps more personally, and many studies have proved its safety and efficiency [2,3,4]. It has been suggested that FS-LASIK behaves as good as or even better than mechanical microkeratomes in the predictability of postoperative spherical equivalent, and seems to have several advantages, but on the other hand no clear evidence of differences between the two modalities in terms of outcomes has been published [5,6 and both early and late flap-related complications have been reported with both approaches [7,8,9,10]. In addition, despite that femtosecond laser was a great technical breakthrough, several special complications have followed up, such as suction loss, rainbow glare, light-sensitivity syndrome, and gas-related complications [4, 11].

**Fig. 1:**
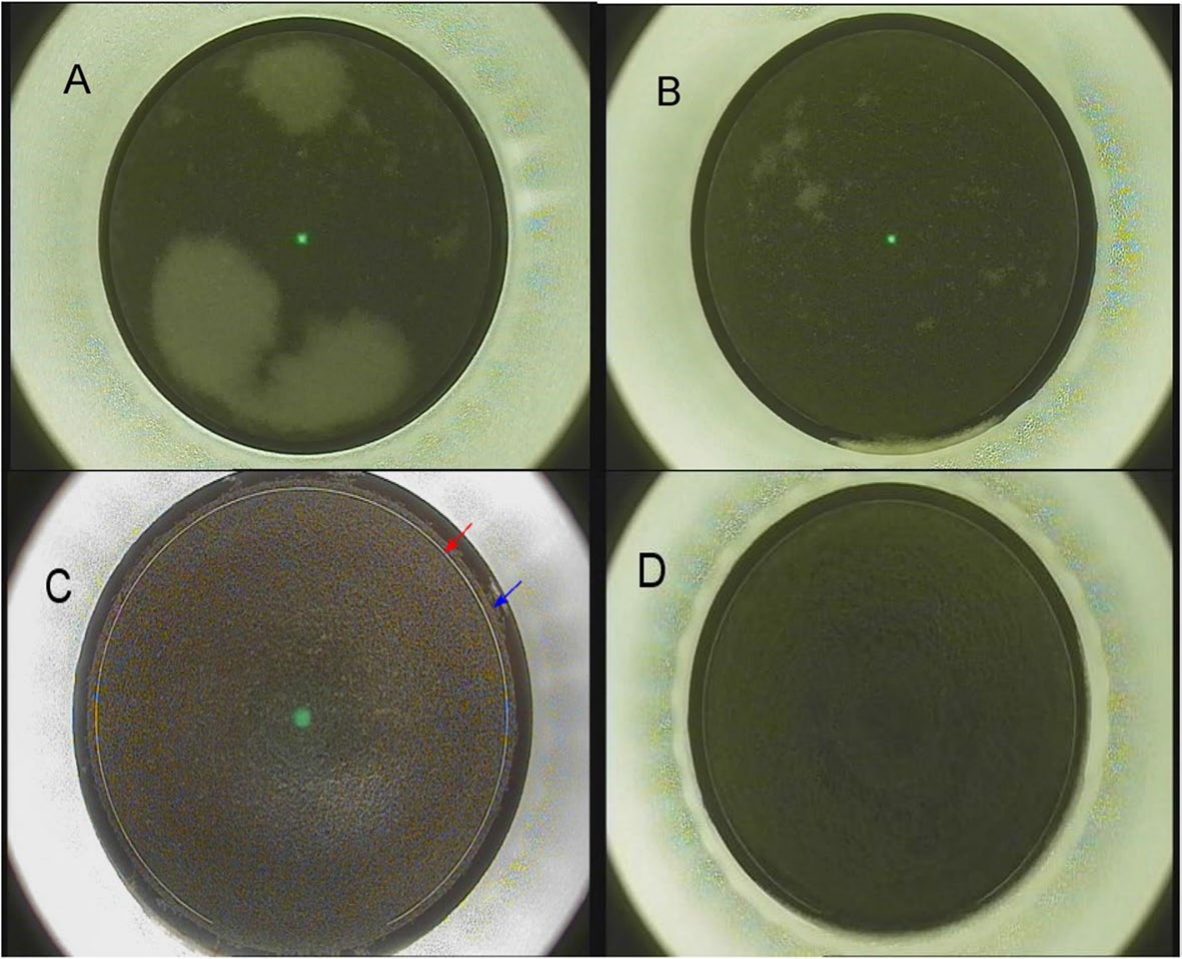
A represented hard OBL.B represented soft OBL.C represented new flap-making pattern, red arrow symbolized flap cut edge, while blue arrow symbolized side cut edge. D represented a traditional pattern in which two edges were overlapping

Opaque bubble layer (OBL) was a relatively common complication during flap cutting, the bubbles were generated from photo-disruptive effect towards corneal tissues which consisted of water vapor and carbon dioxide [4]. When the bubbles are trapped in the stroma, OBL gradually formed. Generally, OBL would not cause severe consequences, however, it could sometimes mislead or obscure laser tracks so that incomplete flap cut might happen and make the flap lifting process more difficult, and tear up the flap. In addition, OBLs might limit the performance of eye tracking and iris-registration technologies as well as subsequent excimer laser ablation [12]. According to the developing speed and appearance, OBL could be divided into hard OBL and soft OBL [13]. Hard OBL forms more quickly than the scanning line of the laser, and it appears denser. While soft OBL generates after the scanning line, it is smaller and diffuser than the hard type (Fig. 1A and B). Hard OBLs posited on scanning line of femtosecond laser or in stroma under the flaps observing by optical coherence tomography [12], however, they would not appear in flaps.

Several studies have reported an OBL incidence ranging from 5 to 73% [13,14,15,16,17] under the Visumax system, with little consistency. Thicker CCT  [13, 15, 18–20] has been considered as a risk factor by most researchers, while other factors such as corneal diameter need further investigations. Besides, the factors influencing the occurrence of the specific type of OBL have not been paid enough attention. Apart from this, a new version of surgical software has been developed recently which created a different way of flap making. We observed that the updated flap-making pattern could apparently generate less OBL clinically than traditional pattern which worth further approving.

Therefore, the purpose of this study was to investigate whether the updated flap-making pattern could really reduce OBL occurrence and investigate the possible risk factors related to the occurrence and type of OBL in LASIK flaps using the Visumax laser system.

### Patients and Methods

This study enrolled myopic/myopic astigmatism patients who underwent FS-LASIK surgeries with Visumax 500 kHz (ZEISS) laser system for flap making process. Inclusion criteria were age 18 years and older, myopia ranging from -1.00 D to -10.00 D, astigmatism less than -3.0 D, corrected distance visual acuity (CDVA) 20/20 or better, and CCT at least 450 μm. Exclusion criteria were: (1) Refraction changed more than 1.00 D in the past year. (2) Any ocular pathology, such as glaucoma, cataract, and corneal ectasia. (3) Any ocular surgical history. (4) Could not understand the risks and benefits of the surgery and refuse to give informed consent. The study was approved by the ethics committee of Tongren Hospital and adhered to the tenets of the Declaration of Helsinki. Informed consent was obtained from each participant.

All patients underwent comprehensive ophthalmic examinations including distance visual acuity (corrected and uncorrected), manifest refraction, cycloplegic refraction, keratometry, corneal topography, CCT, corneal diameter (white to white), and fundus examination. Intraoperative data including flap thickness, ablation thickness, residual stromal thickness, and optical zone were also recorded. Pictures were captured just after flaps creation from videotapes of surgeries. Two investigators (XH, SML) identified OBL’s occurrence as well as its type independently, and any controversy was solved by discussion. The area ratio of OBLs to flap was calculated with Image J software [21].

### Surgical method

After a standard workflow of sterilization and anesthetization, the patients were asked to stare at a green point, then flaps were created using Visumax 500 kHz femtosecond laser system with usual settings of flap creation (Table 1). Excimer laser ablation process (WaveLight, EX500, Alcon) was performed subsequently.

**Table 1:** Settings for flap creation

Settings	Flap cut	Flap side-cut
Spot distance (μm)	4.5	2.0
Track distance (μm)	4.5	2.0
Scan direction	Spiral in	NA
Scan mode	Single	NA
Energy offset	27	27
Flap diameter (mm)	8.0	NA
Hinge position	90°	NA
Hinge angle	50°	NA
*NA* not applicable		

During the flap creation, two different flap-making patterns were applied and labeled as traditional version and updated version respectively. During the flap cut, the eye might slightly move and by that, an offset between flap cut and flap side cut was created. The same lateral shift could occur when re-treating the side cut after a suction loss. The updated version introduced a new parameter allowing to oversize the flap cut diameter in relation to the side cut, and this ‘diameter offset’ could be set in the range from 0 to 0.3 mm in 0.05 mm steps (Fig. 1 C and D). In this study, we chose to set the value at 0.3 mm.

### Statistical analysis

All statistical analyses were performed with SPSS 22.0. The Mann–Whitney test was used for comparisons of continuous parameters, whereas the Chi-square test was used for categorical parameters. Parameters with significance in univariate analyses were included in the Logistic regression model for further investigation. A P value of less than 0.05 was considered statistically significant.

## Results

### Population characteristics and incidence of OBL

Between April 2020 and August 2020, a total of 167 eyes (86 patients) were treated. The patients included 59 females and 27 males, with a mean age of 27.5 ± 6.1 years, mean spherical equivalent of -7.07 ± 2.42 D, and mean astigmatism of -1.03 ± 0.90 D. During the procedure of flap creation, OBLs were observed in 54 eyes (32.3%), consisting of 31 (18.6%) as hard OBLs and 23 (13.8%) as soft OBLs (Fig. 1 A and B). The eyes that produced hard OBLs merged a small amount of soft OBLs. In addition, the mean area ratio of hard OBLs to flap was significantly larger than soft OBLs (14.3±8.3％ vs. 1.1±1.8％, *P*<0.001). 

### Risk factors for OBLs

As shown in Table 2, eyes with OBLs had significantly more myopia than eyes without OBL (− 8.03 D vs. − 6.63 D, *P* < 0.001), CCT was significantly thicker in the OBL group (534 μm vs. 513 μm, *P* < 0.001). In addition, we have investigated whether flap-making patterns could affect OBLs occurrence, eyes adapted new flap-making pattern had lower OBL incidence than the traditional pattern (13.8% vs. 52.5%, χ^2^ = 28.54, *P* < 0.001).

**Table 2:** Characteristics of patients between OBL and OBL-free groups

**Parameters**	**Mean (SD)**		**P**
	**with OBL(*n*** **= 54)**	**without OBL(*n*** **= 113)**	
Age (y)	27.37 (6.07)	27.61 (6.09)	0.65
SE (D)	− 8.03 (1.12)	− 6.63 (2.43)	** < 0.001**
Ast (D)	− 1.09 (0.83)	− 0.99 (0.93)	0.33
AvgK (D)	43.87 (1.28)	43.87 (1.53)	1.00
CCT (μm)	534.04 (33.55)	513.69 (29.79)	** < 0.001**
WTW (mm)	11.89 (0.37)	11.91 (0.38)	0.74
Flap (μm)	101.94 (4.96)	103.72 (14.80)	0.72

*SE*  spherical equivalent, *Ast* astigmatism, *AvgK* average corneal curvature, *CCT* central corneal thickness, *WTW* white to white/ corneal diameter, *Flap*  flap thickness

Logistic regression further confirmed that traditional flap-making pattern (OR = 6.5, 95%CI 3.0–14.3, *P* < 0.001), more myopia (OR = 1.3, 95%CI 1.1–1.5, *P* = 0.01) and thicker CCT (OR = 1.01, 95%CI 1.001–1.03, *P* = 0.04) were associated with a higher risk of incidence of OBL.

As all the hard OBLs came from eyes with the traditional flap-making pattern, it was obvious that eyes with the traditional pattern were prone to develop hard OBLs than the new pattern (73.8% vs. 0%, χ^2^ = 20.8, P < 0.001). Furthermore, eyes with hard OBLs had larger corneal diameters compared with those with soft OBLs (*P* = 0.004), while there were no statistical differences in other parameters between the two types of OBLs (Table 3). Logistic regression revealed that corneal diameter was still a significant risk factor for OBL occurrence (OR = 16.1, 95%CI 1.3–196.4, *P*=0.03).

**Table 3:** Characteristics of patients with hard OBLs and soft OBLs

	**Mean (SD)**		
**Parameters**	**Hard (*n*** **= 31)**	**Soft** **(*n*** **= 23)**	***P***
Age (y)	27.25 (6.56)	27.52 (5.48)	0.61
SE (D)	− 7.71 (2.19)	− 8.47 (1.99)	0.27
Ast (D)	− 1.17 (0.95)	− 1.00 (0.64)	0.96
AvgK (D)	43.66 (1.34)	44.14 (1.16)	0.14
CCT (μm)	538.71 (34.65)	537.74 (31.66)	0.24
WTW(mm)	12.03 (0.34)	11.70 (0.34)	**0.004**
Flap (μm)	102.09 (5.55)	101.74 (4.16)	0.67

*SE* equivalent sphere, *Ast* astigmatism, *AvgK* average corneal curvature, *CCT* central corneal thickness, *WTW*  white to white/ corneal diameter, *Flap*  flap thickness

## Discussion

The current study found that a new flap-making pattern in FS-LASIK, by creating offset between flap-cut and side-cut, was a protective factor for reducing OBLs, while more myopia and thicker CCT were risk factors for developing OBL. Additionally, eyes with hard OBLs seemed to have larger corneal diameters than those with soft OBLs.

The OBL incidences reported in literatures on the Visumax laser system varied from 5% to 72.6%. Our study came up with 32.3% which was comparable to that of a study by Mastropasqua et al. (33.3%), [16], and a study by Wu et al. (28.8%). [15] In South Korea, Jung et al. [13] reported a lower OBL incidence of 5%, whereas Lim et al. reported a much higher incidence of 72.6%. [17] In our study, the hard OBLs accounted for 18.6% of all subjects, smaller than the 28.8% observed by Wu and colleagues [15]. Flap creating processes were all conducted with Visumax 500 kHz laser system in studies mentioned above to make comparisons. The new pattern of flap creation by creating offset between flap-cut and side-cut might account for the lower hard OBL incidence. Besides, the criteria to determine OBLs was subjective to some extent, which might also be one reason for the differences in incidence reported.

The reason for the new flap-making pattern reducing OBL occurrence was that the excessive diameter during flap cutting could be regarded as creating a channel encompassing the flap, as the position of side cuts didn’t change and the flap size remained as designed. When cavitation bubbles were produced, they smoothly diffused into the channel obeying the direction of pressure gradient, from higher pressure to lower pressure, which avoided bubbles’ accumulation. WaveLight FS200 was another commonly used commercial femtosecond laser system, which could create a canal in the hinge of a flap as a pathway for gas [22, 23], while the Intralase system makes a pocket to temporarily store the gas [24]. Despite both systems having unique designations to reduce the incidence of OBL, a relatively lower frequency raised the probability for developing OBL mostly more than 50% [19, 21, 24]. To some extent, it could be concluded that a high-frequency laser system would be a better choice when further combined with a gas diffusing pathway.

Several risk factors might impact the formation of OBLs. Our study found that thicker CCT was associated with a higher OBL incidence, which was consistent with previous studies [13, 15]. This finding could be explained by thicker CCT having more resistant and compact corneas which would increase the obstruction of escaping bubbles. We found that more myopia might be another risk factor for OBLs. To our knowledge, it seems that no study has ever reported a similar result in FS-LASIK. It might be because eyes with more myopia had relatively thinner flaps. However, Li [25] reported that the risk of OBL significantly decreased in eyes with increasing myopia in small incision lenticule extraction (SMILE) which might associate with a deeper scanning plane. Thus, if SE indeed a risk factor cannot be verified now, perhaps it is just the presentative of other factors.

We found that eyes with larger corneal diameters tended to develop hard OBLs. A previous study found that smaller corneal diameter tended to increase overall OBL incidence regardless of OBL types. The given explanation was that a relatively smaller area was outside the suction cone due to the smaller corneal diameter which would impede gas to escape from the high-pressure area (within the cone) to the low-pressure area (outside the cone) [15]. The location of suction cones was nearer to the corneal apex in eyes with the larger corneal diameter as the cones were of the same size. Corneal curvature would gradually become flattered from apex to periphery which meant that eyes with larger corneal diameter would have a steeper contact with the cones [26], and the gas might be difficult to get out from the edge.

In a recent study, Brar et al. [27] found that small contact glass could ease OBL occurrence versus medium size during SMILE, for larger size would increase sagittal height and result in relatively steeper fit which could obscure bubble escaping and cause more severe OBL. In brief, a steeper fit between suction cone and cornea might increase the occurrence of OBL. The effect of corneal diameter on OBL incidence and its mechanism should be investigated in more studies.

In our study, Image J software was used for area calculations of OBL. Soft OBL only took up about 1% of the flap area, while hard OBL was 14% in the current study, both less than the results reported by Liu and coworkers (7.4 ± 5.6%, 28.6 ± 10.1% respectively) [18]. However, they concluded that hard OBLs covered a larger area of flaps when applying an Intralase laser system (60 kHz) for flap creation. Because of such a small area of soft OBLs presented in the current study, it was difficult to precisely circle the image using the Wand tool of Image J software, and the data should be considered more carefully.

There were some limitations in our study. First, despite the current study having a prospective design, it was not a randomized controlled trial so that some confounding factors might bias the results. Second, postoperative visual acuity and visual quality were not evaluated, although several studies reported no significant influence of OBL on postoperative visual acuity [13, 17, 19].

In conclusion, our results revealed that a new flap-making pattern in FS-LASIK, could reduce OBL occurrence by enlarging flap cut diameters and creating a pathway for gas bubbles. It would be more effective if this ‘diameter offset’ is at maximum (0.3 mm) in theory which promises a larger outflow space. Myopic subjects with thicker corneal thickness and larger corneal diameters were more likely to suffer from OBLs during flap creation using Visumax laser, and eyes with larger corneal diameter were also a risk factor for developing hard OBLs which should be studied further.

## Data Availability

The datasets used and/or analysed during the current study available from the corresponding author on reasonable request.
